# Effect of probiotics intake on constipation in children: an umbrella review

**DOI:** 10.3389/fnut.2023.1218909

**Published:** 2023-09-01

**Authors:** Mingyang Dong, Yuting Wu, Mengzhen Zhang, Pengjun Chen, Zhiyang Zhang, Shu Wang

**Affiliations:** ^1^Department of Pharmacy, Shengjing Hospital of China Medical University, Shenyang, China; ^2^Department of Clinical Pharmacy, Shenyang Pharmaceutical University, Shenyang, China

**Keywords:** probiotics, constipation, functional constipation, children, umbrella review

## Abstract

Based on existing systematic reviews and meta-analyse we conducted this comprehensive review to evaluate the quality, effectiveness, and bias of evidence regarding the relationship between probiotic intake and improved constipation outcomes in children. A total of nine meta-analyses and systematic reviews were extracted from 628 articles, summarizing seven effectiveness indicators and the incidence of adverse reactions in the treatment of constipation. According to the results, our study revealed that the intake of probiotics in children with FC significantly improved treatment success rate and defecation frequency, while decreased the recurrence rate of constipation. However, no significant association was detected between probiotics intake and frequency of abdominal pain, stool consistency, frequency of defecation pain, frequency of fecal incontinence of children with FC. The intake of probiotics did not increase the incidence of adverse reactions and demonstrated good safety.

## Introduction

Constipation is a common childhood symptom, with functional constipation (FC) accounting for over 95% of all constipation cases (1). According to the latest diagnostic standard of the Rome IV criteria, FC is characterized by difficulty in defecation, reduced frequency of defecation, or a feeling of incomplete defecation (fecal incontinence), accompanied by abdominal pain, abdominal distension, etc. (2). With an increased fat intake and changes in people’s dietary structure, the prevalence of constipation in children has gradually increased. According to statistics, the global total prevalence of FC in children is about 9.5% (3). The occurrence of FC is related to various factors, including dietary fiber intake, water intake, the level of physical activity, and defecation training (1, 3). Functional constipation affects children’s lives and health. Currently, the etiology and pathogenesis underlying FC have not been elucidated in clinical studies, and there is no consensus on its treatment. Functional constipation not only causes distressing physical, emotional, and social effects in affected children but also negatively affects their families, increasing their medical burden and ultimately damaging the health-related quality of life of the child (2, 4). Relevant studies have also shown that about half of children with constipation remain symptomatic into adulthood, which has a serious impact on their education and daily life, and it has caused significant health problems (5). Therefore, new and more effective treatments for constipation in children are very important for healthy development in childhood.

The gut microbiota is crucial to human health, and its condition is related to many childhood diseases (6). Probiotics are a combination of living bacteria and yeast, including the well-known strains of *Lactobacillus acidophilus*, *Bifidobacterium lactis*, and *L. brevis* (4, 7). Probiotics are now defined as live microorganisms that, when administered in adequate amounts, confer a health benefit on the host by the Food and Agriculture Organization of the United Nations and the World Health Organization (FAO/WHO) (8). This definition is inclusive of a broad range of microbes and applications, while capturing the essence of probiotics (microbial, viable and beneficial to health). The distinction between commensal microorganisms and probiotics is also inferred from this definition. Although commensals in the gut are often the source of probiotic strains, until these strains are isolated, characterized and a credible case presented for their health effects, they cannot be called ‘probiotics’ (8). The use of probiotics the probiotic may change the gut microbial composition and structure and thus can be applied to the clinical treatment of constipation, diarrhea, irritable bowel syndrome, etc., (9). The beneficial effects of probiotics on the intestine may be related to the following aspects: improving gastrointestinal peristalsis and decreasing intestinal transit time; competing with other harmful microorganisms in the intestine for nutrition; adhering to endothelial cells, preventing pathogens from entering the body through intestinal epithelial cells, and stimulating phagocytosis through lymphocyte activation (9, 10). Furthermore, probiotics can stimulate the production of cytokines (such as IgA and INF) and regulate cellular and humoral immunity, thereby affecting the body’s specific and non-specific immunity (2, 11). Their metabolites can lead to a decrease in intestinal PH and the acidification of intestinal contents, thus inhibiting the growth of certain pathogens (12).

Although probiotics can regulate intestinal microbiota and the microenvironment of the intestine, some studies suggest that currently there is insufficient evidence to support the use of probiotic for the treatment of FC in children (13, 14). Clinical randomized double blind controlled trials have shown insufficient evidence to support the use of probiotics for the treatment of FC in children (15–17). However, some studies suggest that the use of probiotic preparations is effective for children with FC. The randomized double-blind controlled trials of *Lactobacillus* and *Bifidobacterium* suggest effectiveness in treating chronic constipation in children (18, 19). A prospective, multicenter study conducted suggests that probiotic formulations can promote average daily bowel movements (20). Therefore, probiotic preparations have an improvement effect on FC in children theoretically. However, there is still controversy internationally about whether probiotic preparations are needed for children with FC. Therefore, we conducted this study to comprehensively review the association between probiotic intake and constipation improvement in children (as reported in our systematic review and meta-analysis) and assess the effectiveness of the existing evidence.

## Materials and methods

### Search strategy

We searched systematic reviews and meta-analyses of observational studies and interventional studies from the databases of PubMed, Embase, Cochrane Library, and China National Knowledge Infrastructure from their inception to January 6, 2023. The search strategy used a combination of the following terms: constipation AND probiotic, prebiotics, synbiotics*, Lactobacillus, L. GG, L. acidophilus, L. rhamnosus, L. plantarum, L. casei, L. gasseri, L. reuteri, L. lactis, Bifidobacterium, B. breve, B. longum, B. infantis, B. adolescentis, B. lactis, Bacillus, Clostridium butyricum, Streptococcus thermophilus, Escherichia coli, Propionibacterium freundendsreichii, Enterococcus SF68, Enterococcus SF68, Enterococcus faecalis, Saccharomyces boulardi,* and *VSL#3* AND systematic review, meta-analysis and review. No restrictions or filters were applied to the search process. We also manually searched the cited references of the retrieved articles and reviews. Two authors (DMY and WS) independently conducted the literature search. Any disagreements between the two researchers in terms of article selection were resolved by a third investigator (CPJ). Details of the search strategy are provided in Supplementary Table S1.

### Eligibility criteria

Meta-analyses and systematic reviews evaluating probiotic intake and constipation in children with outcomes in children were included regardless of the race, gender, country, or region of the participants. Based on these studies, indicators of effectiveness and safety were summarized. If two or more constipation outcomes existed in a single article, the data of each outcome were extracted separately. If one outcome was assessed in more than one study, articles with the largest number of participants were included. Research with unrelated research content, design that was not meet standards, or incomplete research data were excluded.

### Data extraction

Two authors (DMY and WYT) independently extracted data, and disagreements were resolved by consensus. The following data were extracted from the eligible studies: name of the first author; journal; year of publication; type of comparisons; type of studies included (RCT, or non-RCT); number of primary studies; follow-up time; number of participants in each study; estimated summary effect and corresponding 95% confidence intervals (CIs); outcome. Any difference was resolved by the third investigator (CPJ).

### Assessment of methodological quality of included studies and quality of evidence

The methodological quality of each article was evaluated based on AMSTAR-2 (A Measurement Tool to Assess Systematic Reviews 2) items, which is a reliable strategy for evaluating the quality of systematic reviews and meta-analyses (21). The GRADE (Grading of Recommendations, Assessment, Development, and Evaluation) approach was used to evaluate the strength of evidence for each outcome proposed in the meta-analysis and to classify the evidence into “high,” “moderate,” “low,” and “very low” quality to enable recommendations to be made (22).

### Statistical analysis

We extracted data on probiotic intake for improving constipation in children and estimated the overall efficacy using the 95% CI reported in each study (if available). Heterogeneity between studies was assessed using *I*^2^ statistics. Values <50% indicated acceptable heterogeneity, values >50% suggested moderate heterogeneity, and values >75% were indicative of high heterogeneity (23). Egger’s regression asymmetry test was used to calculate an estimate of publication bias for any re-analysis that included at least 10 studies, which was considered indicative of small-study effects. A value of *p* < 0.1 was considered statistically significant according to Egger’s test (24).In addition, *p* < 0.05 was regarded as significant for other tests.

## Results

### Characteristics of the included meta-analyses

**Figure 1:**
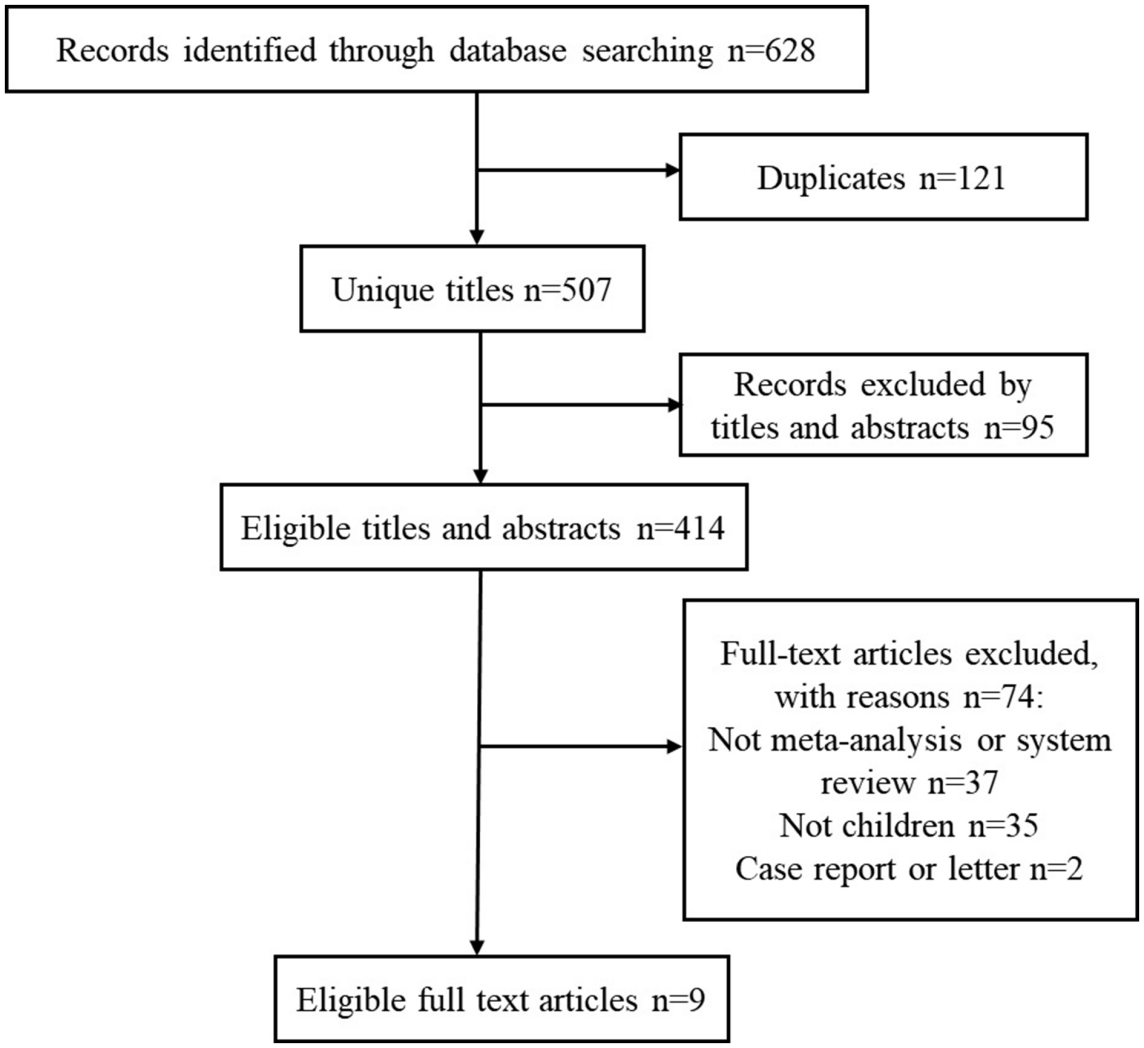
Flowchart of the systematic search and selection process.

The detailed process of literature retrieval and selection is presented in Figure 1. We searched 628 articles and ultimately identified nine meta-analyses based on the inclusion and exclusion criteria. The effectiveness and safety indicators of using probiotics in children with constipation were summarized. Seven indicators of constipation were used as a reference for evaluating the effectiveness of probiotic therapy and were extracted from all eligible studies. In terms of safety, information on whether probiotics increased the incidence of adverse reactions in children was extracted. The effectiveness and safety results of probiotic intake in children with constipation are shown in Table 1. The median number of primary studies was six (interquartile range: 2–7), and the median number of cases was 467 (interquartile range: 111–467).

**Table 1:** The methodological quality of included meta-analysis using AMSTAR-2.

Source	Item 1	Item 2	Item 3	Item 4	Item 5	Item 6	Item 7	Item 8	Item 9	Item 10	Item 11	Item 12	Item 13	Item 14	Item 15	Item 16	Overall quality
Junli Wang (25)	Y	PY	Y	PY	Y	Y	Y	Y	Y	N	Y	Y	Y	Y	Y	N	Moderate
Hao Li (26)	Y	PY	Y	PY	Y	Y	Y	Y	Y	N	Y	Y	Y	Y	Y	N	Moderate
Tabbers (3)	Y	PY	Y	PY	Y	Y	N	Y	N	N	Y	N	N	Y	N	N	Critically Low
Wojtyniak (27)	Y	Y	Y	PY	Y	Y	Y	Y	Y	Y	Y	Y	Y	Y	Y	Y	High
Jin (28)	Y	PY	Y	Y	Y	Y	Y	Y	Y	N	Y	Y	Y	Y	Y	N	Moderate
Gomes (29)	Y	PY	Y	Y	Y	Y	Y	Y	N	Y	Y	N	N	Y	N	Y	Critically Low
Huang (30)	Y	PY	Y	PY	Y	Y	Y	Y	Y	Y	Y	Y	Y	Y	Y	Y	High
Wegh (31)	Y	PY	Y	PY	Y	Y	Y	Y	Y	N	Y	Y	Y	Y	Y	N	Moderate
Chmielewska (32)	Y	PY	Y	PY	Y	Y	Y	Y	N	N	Y	Y	N	Y	N	N	Critically Low

Rationale for selection of items:

1. Did the research of questions and inclusion criteria for the review include the components of PICO?.

2. Did the report of the review contain an explicit statement that the review methods were established prior to the conduct of the review and did the report justify any significant deviations from the protocol?.

3. Did the review authors explain their selection of the study designs for inclusion in the review?.

4. Did the review authors use a comprehensive literature search strategy?.

5. Did the review authors perform study selection in duplicate?.

6. Did the review authors perform data extraction in duplicate?

Di7. d the review authors provide a list of excluded studies and justify the exclusions?.

8. Did the review authors describe the included studies in adequate detail?.

9. Did the review authors use a satisfactory technique for assessing the risk of bias (RoB) in individual studies that were included in the review?.

10. Did the review authors report on the sources of funding for the studies included in the review?.

11. If meta-analysis was performed, did the review authors use appropriate methods for statistical combination of results?.

12. If meta-analysis was performed, did the review authors assess the potential impact of RoB in individual studies on the results of the meta-analysis or other evidence synthesis?.

13. Did the review authors account for RoB in primary studies when interpreting discussing the results of the review?.

14. Did the review authors provide a satisfactory explanation for, and discussion of, any heterogeneity observed in the results of the review?.

15. If the performed quantitative synthesis did the review authors carry out an adequate investigation of publication bias (small study bias) and discuss its likely impact on the results of the review?.

16. Did the review authors report any potential sources of conflict of interest, including any funding they received for conducting the review?.

Y, yes; N, no; PY, partial yes.

### Description and summary of associations

The associations analyzed included seven clinical effect outcomes and one adverse event. The effectiveness indicators of probiotic preparations in the treatment of FC in children were reported in the nine included articles and included the following: treatment success rate, defecation frequency, frequency of abdominal pain, stool consistency, frequency of defecation pain, frequency of fecal incontinence, and recurrence rate. The incidence of adverse reactions was analyzed in terms of safety (Table 1).

### Treatment success rate

Treatment success is usually defined as defecation ≥3 bowel movements per week. According to six studies (3, 26, 27, 29, 31, 32), probiotic preparations could significantly improve the level of treatment success compared with placebos or other interventions. There was significant difference in the treatment success of probiotics and placebos (OR = 4.81, 95% CI: 2.32–9.97, *p* < 0.0001) (26). Most of the RCTs are based on single-strain or mix-strain studies. Research has shown that the combination of *Lactobacillus* and *Enterococcus* can improve the success rate of treatment，and the average effective cumulative dose for children under 1 year old needs to reach 1.05 × 10^9^ CFU can improve the success rate of FC treatment in children, while the average effective cumulative dose for children over 1 year old needs to reach 1.89 × 10^9^ CFU (26). Two RCT reported that *L. casei rhamnosus* Lcr35, but not *L. rhamnosus* GG, showed a beneficial effect in children (18, 33).

### Defecation frequency

Seven studies (3, 25, 27–31) showed that the use of probiotics in children with constipation could significantly increase the frequency of defecation. There was significant difference in terms of defecation frequency improvement between probiotic treatment groups and control groups (MD = 0.73, 95% CI: 0.14–1.31, *p* = 0.02), however, there was significant heterogeneity (*I^2^* = 80%, *p* = 0.02). Subgroup assessments showed that Asian children had a significantly higher stool frequency with probiotic treatment (30). The RCTs found that *L reuteri* and *Bifidobacterium longum* could significantly increase the frequency of bowel movements compared with placebo (19, 34).

### Recurrence rate

Only one study showed that compared with the use of a placebo, the administration of probiotics significantly reduced the recurrence rate of FC, and the heterogeneity was considerable (OR = 0.19, 95% CI: 0.05–0.68, *p* = 0.01) (26). Therefore, probiotic treatment may reduce the recurrence rate of functional constipation in children.

### Frequency of abdominal pain

Six studies (3, 25, 27–29, 31) reported different results for reducing the frequency of abdominal pain and improving the symptoms of abdominal pain in children with FC after using probiotics. Tabbers et al. (3) found that probiotics significantly reduced abdominal pain compared with osmotic laxatives (1.9 episodes with probiotics vs. 4.8 episodes with osmotic laxatives; *p* = 0.04). Gomes et al. (29) showed that in the first week after probiotic intervention, a lower frequency (*p* = 0.017) of abdominal pain was found in a treatment group compared with that in a control group. The remaining four studies showed that there was no significant difference in terms of reducing the incidence of abdominal pain between probiotic treatment groups and control groups. There was no significant difference between the control and probiotic groups in the frequency of abdominal pain at the end of the intervention (OR = 1.09, 95% CI: 0.65–1.82) (25).

### Stool consistency

Three studies (29–31) showed that probiotics had no significant effect on improving stool consistency in children. Stool consistency was not significantly different between probiotic groups and control groups (MD = −0.07, 95% CI: −0.21–0.06, *p* = 0.27) (30).

### Frequency of defecation pain

Three studies (28, 29, 31) showed that compared with the results in a control group, the symptoms and frequency of defecation pain in children in a probiotic group did not improve (RR = 1.16, 95% CI: 0.81–1.66, *p* = 0.41) (28). Pooled results of two RCTs (*n* = 108) (5, 33) showed no difference between the *L. casei rhamnosus Lcr35* and control groups in the frequency of abdominal pain (RR = 2.08, 95%: CI 0.19–23.37), heterogeneity was considerable (*χ*2 = 18.16; *p* < 0.0001; *I*^2^ = 94%) (18, 33).

### Frequency of fecal incontinence

The frequency of fecal incontinence was reported in three studies (27–29). There was no significant effect with probiotics compared with placebos on the frequency of fecal incontinence at the end of intervention (MD = −0.05, 95% CI: −0.63–0.53), and no significant heterogeneity was found (*χ*^2^ = 0.32; *p* = 0.57; *I*^2^ = 0%) (27). Based on the pooled results of two RCTs, there was no significant effect of *L. casei rhamnosus Lcr35* compared with placebo on the frequency of fecal incontinence at the end of intervention (MD = 0.05, 95% CI: −0.63–0.53); no significant heterogeneity was found (*χ*2 = 0.32; *p* = 0.57; *I*^2^ = 0%) (18, 33).

### Adverse reaction rate

Four studies (3, 27, 28, 32) showed no significant difference in the incidence of adverse reactions in the digestive system between probiotic treatment groups and control groups. Chmielewska (32) demonstrated that the probiotics formulation used was tolerable in children, and no adverse event related to it was reported in any trial.

Adverse events were similar in the experimental and control groups (RR 0.58, 95% CI 0.25 to 1.31). No significant heterogeneity was found (*χ*^2^ = 1.01; *p* = 0.6; *I^2^* = 0%). The most frequently occurring adverse events were abdominal pain, vomiting, and gastroenteritis.

### Heterogeneity and publication bias of included studies

Of all 8 associations, 4 had acceptable heterogeneity (<50%), and another 4 had significant heterogeneity (>50%). Four studies of included studies were reported to have significant publication bias, whereas this was not detected in the other five studies.

### Methodological quality

The AMSTAR 2 results for each study are presented in Table 2. Among the nine studies, four (44.4%) were identified as medium in the methodological quality assessment, and three were identified as extremely low [two studies (22.2%)] or low [one study (11.1%)]. The most common key flaws were the absence of detailed literature exclusion lists and funding sources and the failure to consider the risks of bias and heterogeneity when preparing conclusions and recommendations. Evidence of frequency of fecal incontinence and adverse events incidence showed “high” quality according to the GRADE classification, and the others were classified as “moderate” quality. No “low” quality was observed.

**Table 2:** Associations between probiotics intake and constipation in children outcome.

Outcome	Source	Year	No.of primary studies	Duration	No. of cases/total	Metric	Estimates	95%CI	*value of p*	*I^2^*	GRADE rating	AMSTAR-2 rating
***Significant associations***												
Treatment success	Li	2016	9	2–8 wks	345/67	OR	4.81	2.32–9.97	<0.0001	66%	Moderate	Moderate
Defecation frequency	Huang	2017	6	3–12 wks	231/444	MD	0.73	0.14–1.31	0.02	84%	Moderate	High
Recurrence rate	Li	2016	4	2–8 wks	144/243	OR	0.19	0.05–0.68	0.01	62%	Moderate	Moderate
***Non-significant associations***												
Stool consistency	Huang	2017	3	3–8 wks	133/267	MD	−0.07	−0.21-0.06	0.27	92%	Moderate	High
Frequency of abdominal pain	Wang	2014	3	3–8 wks	152/293	OR	1.09	0.65–1.82	0.75	0%	Moderate	Moderate
Frequency of defecation pain	Wang	2014	2	3–8 wks	108/209	OR	1.16	0.79–2.72	0.23	0%	Moderate	Moderate
Frequency of fecal incontinence	Wojtyniak	2017	5	3–12 wks	212/462	MD	−0.05	−0.63-0.53	0.57	0%	High	High
Adverse events	Wojtyniak	2017	6	3–12 wks	234/462	RR	0.58	0.83–1.62	0.6	0%	High	High

## Discussion

Probiotics are often used in the clinical treatment of constipation in children, and many meta-analyses and systematic reviews of their efficacy have been conducted. On this basis, we conducted an umbrella review to analyze whether probiotics can improve constipation in children. We analyzed a total of nine meta-analyses and systematic reviews and summarized seven indicators of effectiveness in treating constipation. According to the results, our study revealed that the intake of probiotics in children with FC significantly improved treatment success rate and defecation frequency, while decreased the recurrence rate of constipation. However, no significant association was detected between probiotics intake and frequency of abdominal pain, stool consistency, frequency of defecation pain, frequency of fecal incontinence of children with FC. The incidence of adverse reactions was low and the safety was good when consuming probiotics. These results suggest that probiotic intake did improve constipation in children, although they cannot improve some symptoms of constipation in children.

As we know, probiotics can regulate the gut microbiota. Intestinal microecology consists of intestinal microbiota, intestinal epithelial cells, and intestinal mucosal immune system (35). The gut microbiome is comprised of the collective genome of microbes inhabiting the gut including bacteria, archaea, viruses, and fungi, including the genes and genomes of the microbiota, as well as the products of the microbiota and the host environment. Microbiome comprises all of the genetic material within a microbiota (36). The difference between the intestinal microbiota of the population in general may be the cause of constipation. The composition of intestinal microbiota varies from person to person and is affected by a range of factors, including diet, living environment, and drugs (34). And there are also differences among different populations.

The pivotal role of intestinal microbiota in the occurrence and development of constipation has prompted a shift in therapeutic options toward microecological intervention, especially probiotics, which has gradually replaced the traditional approaches for treating constipation (37). Those most widely studies are organisms within the genera *Bifidobacterium* and *Lactobacillus*. There is still controversy internationally about whether probiotics are needed for children with FC, and some research results indicate that probiotic preparations have an improvement effect on children with FC. In the study of probiotic therapy for FC in children, most studies have heterogeneity in terms of study population, probiotic strains, dosage, study duration, and follow-up. Tabbers et al. (15) indicated that both fermented dairy product containing *Bifidobacterium lactis* DN-173010 and control product could improve stool frequency from baseline to after 3 weeks, with no significant difference between both, and that no serious adverse events were observed in children with FC. Wojtyniak et al. (33) suggested that *Lactobacillus casei rhamnosus* was not associated with significant improvement in symptoms in children with FC aged less than 5 years and did not recommend the use of probiotics in children with FC. While the administration of *L. casei rhamnosus* Lcr35 augmented the number of stools and reduced the number of hard stools. Again, although the results were statistically signifcant, the overall effects were clinically modest. All of the conclusions are based on single studies, some of which had a very small number of participants and methodological limitations. The conclusions should be interpreted with great caution. Repeat studies with the probiotic strains that have been proven effective are needed. A paucity of data did not allow us to conclude whether any particular probiotic is more effective than another. In terms of microbial alterations in adults, the analysis of the difference in efficacy of probiotic subgroups shows that *B.lactis* of *Bifidobacterium* can significantly improve the rectosigmoid transit time, defecation frequency, hard stools, flatulence in patients with chronic constipation, while *L.casei Shirota* of *Lactobacillus* has no obvious therapeutic effect (38, 39). It is worth noting that the analysis of this research for different microbiota subgroups has high heterogeneity. However, another meta-analysis of a controlled study on adults with FC found that probiotic therapy can significantly improve symptoms in patients with FC, but the impact of *B. lactis* on treatment efficacy is not significant. In addition, the study also pointed out that increasing the variety of microbiota can significantly improve the symptoms of FC (40). This suggests that multi-strain probiotic mixtures may be more effective in treating FC than single-strain probiotic. Due to the differences in function and immunogenicity of the microbiota, the impact on the host may not be the same among different strains of the same species, and there are significant differences in the therapeutic effects of different strains of the same species on FC.

There are significant differences in the dosage of probiotic therapy among various researches. A RCT targeting constipation patients administered two different doses of *B. lactis* and placebo, after 2 weeks of treatment, probiotics showed a dose-dependent reduction in the whole gut transit time compared to placebo, indicating that high-dose probiotic therapy is more beneficial for constipation patients (41).The results of probiotic treatment are based on occupancy effects, only when a sufficient dose of probiotics is given can the beneficial microbiota be effectively restored. In addition, due to the limited colonization sites in the intestine, excess probiotic cannot be fully colonized. Therefore, even if excessive probiotic treatment is used, its beneficial effects on host health are very limited. There are alsodifferences in the treatment course of probiotics for FC among different researches, ranging from 2 to 12 weeks. The current research on whether different intervention times have an impact on the effectiveness of probiotics in treating FC is not clear, which requires further research to answer.

The gut microbiota may be involved in the production or aggravation of constipation. In adults, experimental studies have shown that constipation is often associated with gut microbiota dysbiosis, consisting of the modified abundance of certain taxa of the colonic microbiome (39). For example, some data have suggested the decreased abundance of *Bifidobacteria, Lactobacillus, Bacteroides,* and *Prevotella* (42). Research has shown that there is an increasing trend in the relative abundance of *Bifidobacteria* and *Lactobacilli* in adolescent FC patients (43). In children, one recent study showed that in those with FC, the most discriminative species were *Bacteroides fragilis, Bacteroides ovatus, Bifidobacterium longum, Parabacteroides* species (increased), and *Alistipes finegoldii* (decreased) (44). Although research has found that probiotics do not significantly alleviate symptoms of FC in children, meta-analysis of adult FC patients has shown that probiotics treatment can effectively reduce the whole gut transit time and rectosigmoid transit time, increase defecation frequency, reduce difficulty with evacuation, bloating, abdominal pain or discomfort and hard stools (38). Therefore, we speculate that the difference in efficacy may be related to different microecological imbalances in adult and child FC patients. Studies have shown that probiotics have a certain role in diseases such as diarrhea, constipation, inflammatory bowel disease, and irritable bowel syndrome (45, 46). Probiotics can effectively regulate multiple aspects of the pathogenesis of FC. First, probiotics can regulate the composition of the gut microbiota, which can be divided into three categories based on physiological functions: 1. physiological microbiota, mainly including obligate anaerobes, such as *Bifidobacterium and Lactobacillus,* which are the dominant gut microbiota and play a protective role in the normal physiological function of the intestine; 2. opportunistic pathogens, including *E. coli, Enterococcus,* and *Clostridium*, are mostly obligate anaerobes that only affect the host under certain special conditions; and 3. pathogenic bacteria, mostly *Proteus*, can cause human diseases if they are colonized in the intestinal tract for a long time and multiply in large numbers (47–49). On the one hand, probiotics compete for nutrients to produce metabolites and soluble factors, such as lactic acid, short-chain fatty acids (bacteria), and hydrogen peroxide, which affect the growth of pathogenic bacteria. On the other hand, by promoting the production of mucoprotein and reducing the adhesion of pathogenic bacteria, probiotics have a protective effect on pathogens and improve the intestinal ecosystem (50, 51). Second, constipation is associated with impaired intestinal motility and gas retention, while probiotics may improve irregular intestinal peristalsis and flatulence (52). The mechanism by which the gut microbiota promotes intestinal peristalsis is not yet clear and may be related to the regulatory effects of probiotics on SCFAs and 5-HT (53, 54). Opportunistic pathogens, such as *Bifidobacterium and Lactobacillus*, can ferment and degrade in the intestine, reduce intestinal pH, and produce metabolites, including SCFAs. Mainly, SCFAs include acetic acid, propanoic acid, butyric acid, etc. (55). SCFAs can reduce intestinal pH, promote colonic peristalsis, and effectively reduce the retention time of feces in the colon. A low-PH environment can also promote the setting of obligate anaerobes in the intestinal tract *via* positive feedback regulation, enabling SCFAs to competitively bind to enterocyte or receptor binding sites on the mucosa (56, 57). This improves colonization resistance, inhibits colonization and reproduction by opportunistic pathogens and pathogenic bacteria, improves intestinal microbiota disorder in patients with constipation, maintains a normal intestinal microbiota structure, and helps prevent and alleviate constipation and symptoms caused by intestinal microbiota disorder (58, 59).As far as we know, the role of probiotics in treating childhood constipation is uncertain due to conflicting research results. Whether probiotics can improve constipation in children is currently controversial; It is worth noting that the present study is the first comprehensive summary and evaluation based on the available evidence as to whether probiotic intake can improve constipation in children. Standard tools were used to assess the methodological quality (AMSTAR) and strength of evidence (GRADE) of the included literature (17, 18). However, although methodological patterns were used correctly, selection bias may still exist. To minimize this bias to the greatest extent possible, two authors applied the above methods. According to our systematic analysis of the research, the use of probiotics can significantly improve constipation in children，although probiotics do not significantly improve some symptoms of FC. Moreover, the safety of probiotics is tolerable for children.

Several limitations exist in our study. First, only two of the included studies were classified as high quality according to the AMSTAR-2 method due to most meta-analyses being based on observational studies. Second, differences in the type, dosage, and duration of probiotic use may have affected the research results. Third, the diet and physical condition of children may affect the therapeutic effect of probiotics. Finally, the simultaneous use of other drugs can also affect the outcome of treatment. In studies on probiotic therapy in children, the sample size is generally not large enough, which may also have affected our conclusions. Considering this study’s shortcomings, further high-quality research on this topic is needed.

## Author contributions

MD and YW conducted this research and wrote the paper. SW and MZ designed the study and had primary responsibility for final content. PC and ZZ provided essential materials and analyzed data. All authors contributed to the article and approved the submitted version.

## Funding

This study was supported by 345 Talent Project of Shengjing Hospital of China Medical University.

## Conflict of interest

The authors declare that the research was conducted in the absence of any commercial or financial relationships that could be construed as a potential conflict of interest.

## Publisher’s note

All claims expressed in this article are solely those of the authors and do not necessarily represent those of their affiliated organizations, or those of the publisher, the editors and the reviewers. Any product that may be evaluated in this article, or claim that may be made by its manufacturer, is not guaranteed or endorsed by the publisher.
