# Supplementary material for: Effect of probiotics intake on constipation in children: an umbrella review
Source: Front Nutr. 2023 Sep 1;10:1218909. doi: 10.3389/fnut.2023.1218909 (PMC10502344; doi:10.3389/fnut.2023.1218909)
Supplement: Supplementary file 1 [file Table_1.docx]

**Supplementary Table 1. The general characteristics of the included meta-analysis**

| Source | Patient | Intervention  group | Control  group | Type of studies included | No.of primary studies | No. of cases | Follow-up  time (wks) | Outcome |
| --- | --- | --- | --- | --- | --- | --- | --- | --- |
| Junli Wang 2014^[33]^ | Children or adolescents aged 2-20 years excluding other organic diseases, and the diagnostic criteria for constipation according to the Roma standards | Probiotics：  *B.longum* (1 x10^9^ CFU/d)   1. *LGG* (2 x10^9^ CFU/d)   *DN-173010* (4 x 10^9^ CFU/d)  *LGG* (10 x10^9^ CFU/d)  *L.casei rhamnosus Lcr35* (8 x 10^8^ CFU/d)  Symbiotic (1 x 10^9^CFU/d) | Placebo | RCT | 6 | 421 | 3-12 | Defecation frequency  Frequency of abdominal pain |
| Hao Li 2016^[29]^ | Children aged 0–18 years with functional constipation diagnosed according to the Rome III | Probiotics:  live combined *Bifidobacterium*，*Lactobacillus* and *Enterococcus* capsules (2-4 tablets bid) | Placebo | non-RCT | 9 | 673 | 2-8 | Treatment success  Recurrence rate |
| Tabbers2014^[3]^ | Children，the diagnostic criteria for constipation according to the Roma III, or＜3 bowel movements per week, or anal fissure caused by hard stool lumps | 1. Probiotics:   *LGG; Lactobacillus casei* DN; *Bifidobacterium lactis* DN; *Bifidobacterium longum*; *L reuteri*  (2) Probiotics:  *L. casei rhamnosus* (8 x 10^8^ CFU/d) | (1) Placebo (2)Laxatives | RCT | 6 | 354 | 4-8 | Treatment success  Defecation frequency  Frequency of abdominal pain  Adverse events |
| Wojtyniak2017^[28]^ | Children aged 0–18 years with functional constipation diagnosed according to the Rome II, III, or IV criteria | Probiotics:  *L. casei rhamnosus Lcr35*  *LGG*  *B. lactis* DN-173010  *L. reuteri* DSM 17938  *B. longum* | Placebo | RCT | 7 | 515 | 3-12 | Treatment success  Defecation frequency  Frequency of abdominal pain  Adverse events |
| Jin2018^[32]^ | Children of a mean age of <16 years, with functional constipation, diagnosed according to clinical symptoms, opinions of pediatricians, or the Rome I, II, or III criteria | (1)Probiotics with Lactulose:  *LGG(*8x10^9^CFU/d )+1mL/kg/d of 70% Lactulose  (2) Probiotics:  *Lcr35* (8 x10^8^ CFU/d) (3) Probiotics:  *B. lactis* DN-173010(4.25 x10^9^ CFU/d) (4) Probiotics:  *Lcr35* (8 x10^8^ CFU/d) | (1) Placebo with Lactulose:  1 mL/kg/d of 70% lactulose plus placebo  (2)MgO(50 mg/kg/d) or Placebo (3) Nonfermented dairy product (125-g pot) without probiotics and with a low content of lactose (<2.5 g per pot) (4) Placebo | RCT | 4 | 382 | 3-12 | Defecation frequency  Frequency of abdominal pain  Frequency of defecation pain  Frequency of fecal incontinence  Adverse events |
| Gomes2020^[26]^ | Children aged 0–18 years with functional constipation diagnosed according to the Rome III (2006) | (1) Probiotics with Lactulose:  *L. rhamnosus* GG *531032*(1x 10^9^ CFU bid) + Lactulose (2) Probiotics:  *L. casei rhamnosus Lcr35*( 8 × 10^8^ CFU bid) (3) Probiotics:  *Lactobacillus reuteri DSM 17938* (1 x 10^8^ CFU bid) (4) Probiotics:  *Bifidobacterium lactis DN-173010*( 4.25 x 10^9^ CFU bid) (5) Probiotics with PEG 4000: probiotic mix: Brief *Bifidobacterium* M-16 V®, Infant *Bifidobacterium* M-63® and *Bifidobacterium longum* BB536® | (1) Placebo with Lactulose (2)MgO/ Placebo (3) Placebo (4) Placebo with Lactulose (5)PEG 4000 | RCT | 8 | 564 | 2-14 | Treatment success  Defecation frequency  Stool consistency  Frequency of abdominal pain  Frequency of defecation pain  Frequency of fecal incontinence |
| Huang2017^[31]^ | Children aged ≤18 years with constipation diagnosed according to clinical symptoms, opinions of pediatricians, or the Rome I, II, or III criteria. | Probiotics:  *Lactobacillus rhamnosus GG* (1x 10^9^CFU 2/d)  *Lactobacillus casei rhamnosus, Lcr35(*1.6 × 10^9^CFU 2/d)  *L. casei,L. rhamnosus, S. thermophilus, B.breve, L.acidophilus, B. infantis*(1 × 10^9^CFU /d)  *Lactobacillus rhamnosus PXN54，Streptococcus thermophiles PXN66，Bifidobacterium breve PXN25,Lactobacillus acidophilus PXN35, Bifidobacterium infantis PXN27, Lactobacillus* *bulgaricus*(1 x 10^9^CFU/d)  *Lactobacillus sporogenes*(15 x 10^7^ spores,1Tab/20 kg/day)  *Lactobacillus delbruec kiissp*. *Bulgaric*us*CNCM strain* *numbers 1-1632* and *1-1519*, *Streptococcus the rmophilus CNCMstrain, Lactococcuscremoris BlactisDN-173 010*(1.2 x 10^8^CFU per pot, two pots per day) | Placebo | RCT | 6 | 498 | 3-12 | Defecation frequency  Stool consistency |
| Wegh2018^[25]^ | Children aged 0-18 years with functional constipation diagnosed according to Rome II, III or IV criteria. | (1) Probiotics:  *Lactobacillus casei rhamnosus Lcr35*  (2) Fermented milk containing probiotics:  *Bifidobacterium lactis DN173 010*  (3) Goat yoghurt containing probiotics:  *Bifidobacterium longum*  (4) Probiotics with Lactulose:  Protexin (5) Probiotics with PEG 4000:  *Bifidobacteria breve M-16 V, Bifidobacteria infantis M-63,* and *B. longum BB536* | (1) Placebo (2) Unfermented milk diary without probiotics (3) Goat yoghurt without probiotics (4) Placebo with Lactulose (5)PEG 4000 | RCT | 6 | 467 | 4-8 | Treatment success  Defecation frequency  Stool consistency  Frequency of abdominal pain  Frequency of defecation pain |
| Chmielewska2010^[27]^ | Children aged 2-16 years with constipation < 3 spontaneous bowel movements per week for at least 12 weeks | Probiotics:  *E. coli Nissle* 1917(25 × 10^9^ CFU/d)  *L. casei Shirota*(6.5 × 10^9^ CFU/d)  *B. lactis DN-173* 010(Fermented milk containing 1.25 × 10^10^ CFU of probiotic plus yoghurt strains)  *L. rhamnosus GG(Lactulose plus LGG* 2 × 10^9^ CFU/d)  *L. casei rhamnosus Lcr35*(8 × 10^8^ CFU/d) | Placebo | RCT | 2 | 111 | 4-12 | Treatment success  Adverse events |
